# Microfluidic model for in vitro acute *Toxoplasma gondii* infection and transendothelial migration

**DOI:** 10.1038/s41598-022-15305-4

**Published:** 2022-07-06

**Authors:** Hyunho Kim, Sung-Hee Hong, Hyo Eun Jeong, Sewoon Han, Jinchul Ahn, Jin-A. Kim, Ji-Hun Yang, Hyun Jeong Oh, Seok Chung, Sang-Eun Lee

**Affiliations:** 1 School of Mechanical Engineering, Korea University, Seoul, Republic of Korea; 2 Center for Systems Biology, Massachusetts General Hospital Research Institute, Boston, MA USA; 3 Division of Vectors and Parasitic Diseases, Korea Diseases Control and Prevention Agency, Cheongju, Republic of Korea; 4 CellFE, Alameda, CA USA; 5 Next & Bio, Seoul, Republic of Korea; 6 KU-KIST Graduate School of Converging Science and Technology, Korea University, Seoul, Republic of Korea

**Keywords:** Biomedical engineering, Parasite biology

## Abstract

The protozoan parasite *Toxoplasma gondii* (*T. gondii*) causes one of the most common human zoonotic diseases and infects approximately one-third of the global population. *T. gondii* infects nearly every cell type and causes severe symptoms in susceptible populations. In previous laboratory animal studies, *T. gondii* movement and transmission were not analyzed in real time. In a three-dimensional (3D) microfluidic assay, we successfully supported the complex lytic cycle of *T. gondii *in situ by generating a stable microvasculature. The physiology of the *T. gondii*-infected microvasculature was monitored in order to investigate the growth, paracellular and transcellular migration, and transmission of *T. gondii*, as well as the efficacy of *T. gondii* drugs.

## Introduction

**Figure 1:**
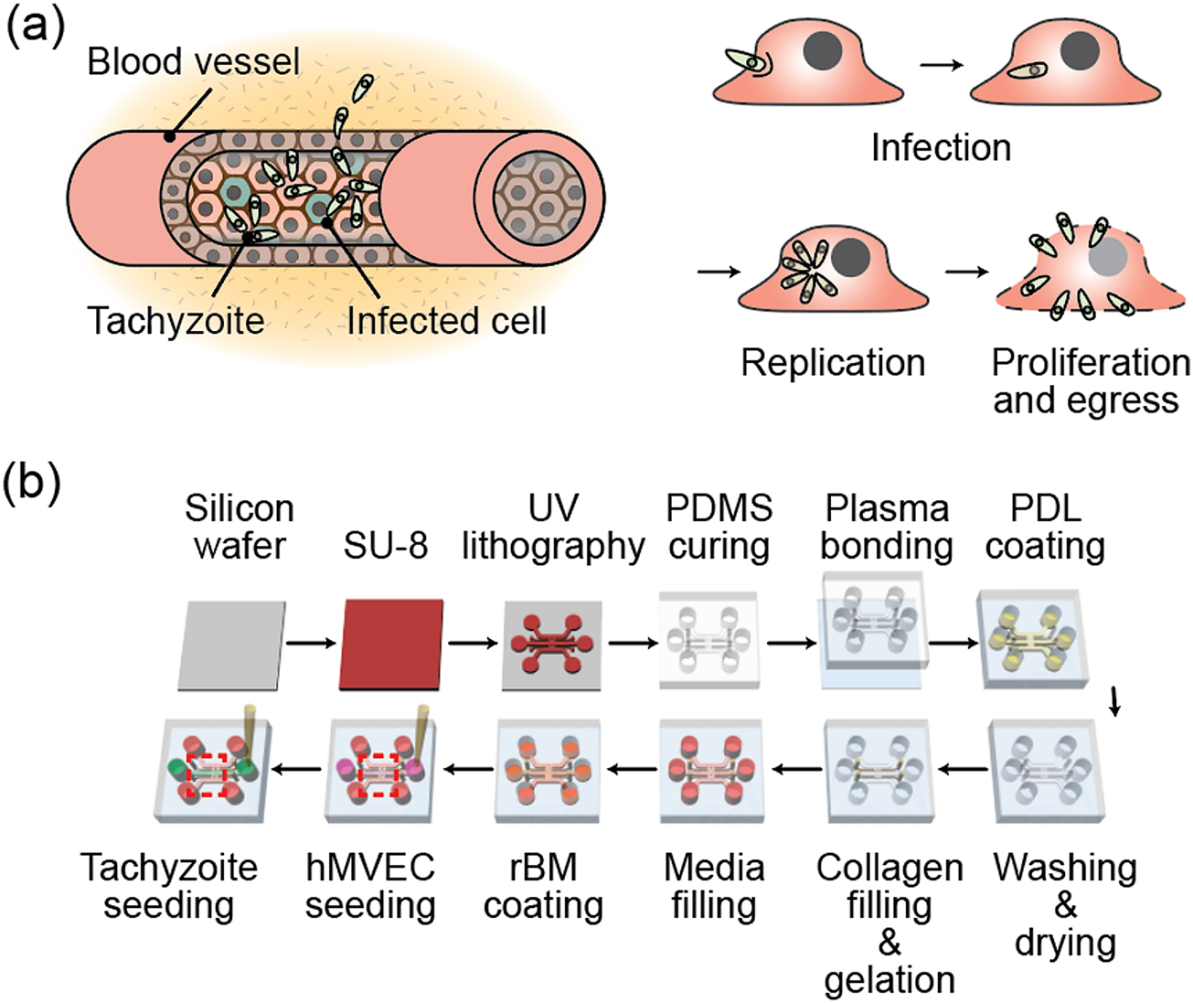
(**a**) Diagrams showing the infection of a blood vessel by a tachyzoite. After penetrating the cell membrane, the tachyzoite relicates inside the cell and then egresses. (**b**) Pictorial representation of the manufacturing process for the microfluidics platform used in the experiment.

One-third of the world's population is infected with *Toxoplasma gondii (T. gondii)*, the causative agent of one of the most prevalent zoonotic diseases^1^. Water, food, and soil contamination are the sources of infection^2,3^. Oocytes or cysts of parasites that have been ingested migrated from the intestine to secondary tissue. The majority of *T. gondii* infected individuals exhibit only mild symptoms. However, as immunity declines in conditions such as acquired immunodeficiency syndrome (AIDS) and organ transplants, disease severity increases. The clinical significance of *T. gondii* cannot be overstated, given that the pathogen has already infected a large number of people around the world^4,5^. *T. gondii* is capable of triggering both acute and chronic infections. The rapidly growing tachyzoites, highly dynamic stage of *T. gondii*, develop into slow-growing bradyzoites in chronically infected tissues^6^. Tachyzoites infect adjacent cells and tissues after entering blood vessels (Fig. 1a). They can cross biological barriers such as the intestinal barrier, the blood–brain barrier, the blood-eye barrier, and the placental barrier, causing severe diseases such as encephalitis^7^ and chorioretinitis^5,8^ and even fetal death^9,10^.

The intricate life cycle of *T. gondii*, which includes infection, proliferation, and dissemination, has been studies in laboratory animals^7,8^. Moreover, although infected animals can be sacrificed to trace the post-infection pathological status of a tissue, the intricate sequences that follow acute infection are still not fully understood^9,10^. Using immune cells such as macrophages^11,12^, dendritic cells^12,13^, microglia^14^, kidney^15^, brain^16,17^, and retina^18,19^, in vitro studies have monitored the infection and interaction between tachyzoites and the two-dimensional (2D) cultured host cells during tachyzoites replication, penetration, and migration. In the endothelial monolayer^20,21^ and epithelial monolayer^22,23^ models, the excretory/secretory proteases (ESPs) of *T. gondii* degrade junction proteins such as ZO-1, Claudin-1, Occludin, and E-cadherin, and increase actin filament redistribution and permeability. However, previous 2D studies do not accurately reflect the physiological microenvironment in during *T. gondii* infection. Alterations in the molecular expression of intercellular adhesion molecule-1 (ICAM-1)^15,18^, parasite adhesion MIC2^15^, and interferon-γ (IFN-γ)^24^ in endothelial cells (ECs) have been revealed in adhesion and cell–cell junction penetration of tachyzoites.

Microfluidic systems have represented physiological fluid flow and microenvironment for *T. gondii* infection research. Lodeon’s Group reported that shear stress reduced the adhesion and motility of *T. gondii* on the EC monolayer^25^*.* Their another paper reported that infection with *T. gondii* affects Hippo and YAP signaling in ECs, thereby remodeling the cytoskeleton, altering permeability and causing junction damage in the EC monolayer^26^. However previous studies only with an EC monolayer on the bottom of a plastic cell culture dish lacked extracellular components below the EC monolayer as well as microenvironmental factors of actual tissues. *T. gondii* can more easily infect EC cells, but cannot migrate across them. The models were incapable of simulating the migration of tachyzoites across the EC monolayer into the extracellular matrix (ECM) and the complex interaction with neighboring cells. Other models with cerebral organoid^27^, organoid derived monolayers^28^, EC beads^29^, and incorporated ECMs (e.g., collagen and Matrigel)^30–32^ have demonstrated *T.gondii*'s interaction with adjacent environmental factors. However, they located *T.gondii* on EC monolayer and made the pathogens penetrate vertically, necessitating the use of a confocal microscope for observation. In this study, we hope to direct pathogen movement and penetration in a horizontal direction through a vertically cultured EC monolayer, thereby providing a clear investigation on the complex *T. gondii* lytic cycle. We used hydrogel incorporating microfluidic platform reconstituting blood vessels to study angiogenesis^33–35^, vasculogenesis^36,37^, drug permeability^26^, and the transendothelial migration of immune^38^ and cancer cells^39^. Protocols were revised to examine tachyzoite migration through the EC monolayer into the ECM, and toward the neurons in order to investigate infection, proliferation, and migration of *T. gondii*, interacting with host cells and the surrounding microenvironment. under a microscope, a lytic cycle of the tachyzoite interacting EC monolayer, ECM and neurons was clearly observed in quantitative manner.

## Materials and methods

### Preparation of GFP-expressing *T. gondii*

The transgenic green fluorescent protein (GFP)-expressing *T. gondii* strain was created by Prof. Yoshifumi Nishikawa (National Research Center for Protozoan Diseases, Obihiro University of Agriculture and Veterinary Medicine, Japan). GFP-positive tachyzoites were kindly provided by Dr. Young-Ha Lee (Chungnam National University School of Medicine, Korea). Tachyzoites was electroporated by PLK strain using transfer vector, plasmid containing DHFR-GRA5′-MCSGRA3′-GRA5′-GFP-GRA3′ fragment^40^. HeLa cells (ATCC, Manassas, VA, USA) were inoculated with GFP-positive tachyzoites at a ratio of 1:3. After 3 days, tachyzoites were harvested from infected HeLa cells and passed twice through a 25-gauge needle and a 5 μm syringe filter (Millipore, USA) to remove cellular debris and host cells. The cells were then washed with Dulbecco’s phosphate-buffered saline (DPBS), centrifuged (3000×*g* for 5 min), and suspended in DPBS.

### Preparation of EC and neuron

Purchased human microvascular endothelial cells (hMVECs) were cultured in complete medium (Lonza, Basel, Switzerland). The hMVECs were used at passages 7 and 8, with a seeding density of 2 × 10^6^ cells/ml. Neurons were isolated from the brain cortex of E15 ICR mouse euthanized by CO_2_ inhalation, according to procedures^41,42^, and were then immediately seeded and cultured. All animal experiments were conducted in accordance with institutional guidelines and protocols approved by Korea University's Institutional Animal Care and Use Committee (KUIACUC-2017-71 and KUIACUC-2022-0028). The study was reported in accordance with ARRIVE guidelines.

### Preparation of the microfluidic assay

An ECM-mimicking hydrogel incorporating microfluidic assay was fabricated using soft lithography of polydimethylsiloxane (PDMS; Sylgard 184; Dow Corning, MI, USA) as described previously^43^. The chip was sealed with a cover glass by using O_2_ plasma (Femto Science, Seoul, Korea). Channels were filled with poly-d-lysine (PDL; Sigma, St. Louis, MO, USA) solution and incubated at 37 °C for 2 h. The PDL solution was washed and aspirated twice with sterile deionized water (DDW) and stored in an 80 °C dry oven until the day of the experimentation. Type I collagen solution was prepared with 10× PBS, NaOH, DDW, and type I collagen (BD Biosciences, CA, USA). The solution was then injected into the hydrogel channel and gelled for 30 min in a 37 °C incubator. After gelation, cell culture medium was filled into the medium channels. Recombinant basement membrane (rBM) was formed on the ECM using a Matrigel (BD Biosciences, CA, USA) coating to form a confluent and stable EC monolayer^44^. After rBM reconstitution, hMVECs were seeded at a density of 2 × 10^6^ cells/ml and cultured for 5 days to form a confluent EC monolayer (Fig. 1b).

### *T. gondii* inhibitors

Genistein (4′,5,7-Trihydroxyisoflavone, a tyrosine kinase inhibitor) and blebbistatin (1-Phenyl-1,2,3,4-tetrahydro-4-hydroxypyrrolo [2.3-*b*]-7-methylquinolin-4-one, a myosin II inhibitor) were dissolved in dimethyl sulfoxide (DMSO) as stock solutions^45,46^. Each stock solution was diluted with culture medium and the final concentration was adjusted to 100 μM. The calcium ionophore, A23817, was dissolved directly in the culture medium^47^. The final concentration of A23187 was 100 µM^46^. All chemicals were obtained from Sigma-Aldrich (St. Louis, MO, USA).

### Imaging and analysis

Fluorescence microscopy (Zeiss, Swiss) was used to follow the infection and migration of tachyzoites. For imaging purposes, cells were fixed using 4% paraformaldehyde and permeabilized with a 0.1% Triton-X100 solution. Tachyzoites were tagged with GFP, and the cytoskeleton of ECs was stained with rhodamine and phalloidin (Invitrogen, MA, USA). 3D images were acquired with confocal microscopy (LSM700, Zeiss, Swiss).

## Results

### *T. gondii* infection and its transmigration to the confluent three-dimensional (3D) EC monolayer

**Figure 2:**
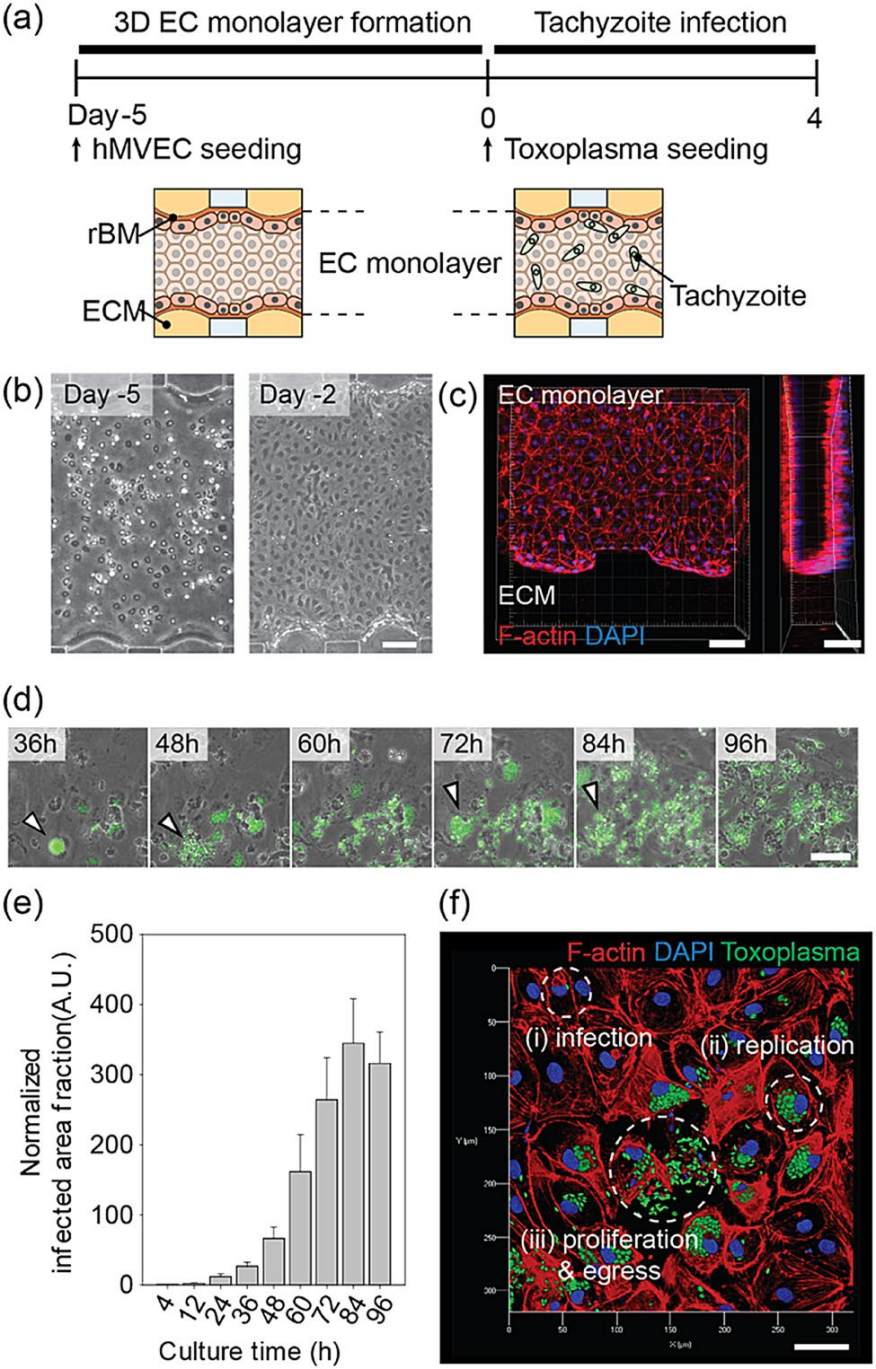
Tachyzoite infection model with a 3D EC monolayer in the microfluidic platform. (**a**) Time line of 3D EC monolayer formation and tachyzoite infection. (**b**) Phase images of EC monolayer at days 5 and 2. (**c**) Confocal image of the 3D EC monolayer on day 0 before seeding the tachyzoites. (**d**) Sequential phase images with 12 h intervals after the infection of tachyzoites. Infected cells indicated by arrowheads show the cycle, from duplication to egress. Scale bar indicates 100 µm. (**e**) Area fraction of tachyzoite in the EC monolayer and ECM region. (n = 8 for each group). (**f**) Overview of the tachyzoite cycle within the EC monolayer. Scale bar indicates 50 μm. Statistical significance was analyzed by one-way ANOVA with Holm-Sidak method (**e**) and is indicated by asterisks as follows: *P < 0.05, **P < 0.01, ***P < 0.001.

**Figure 3:**
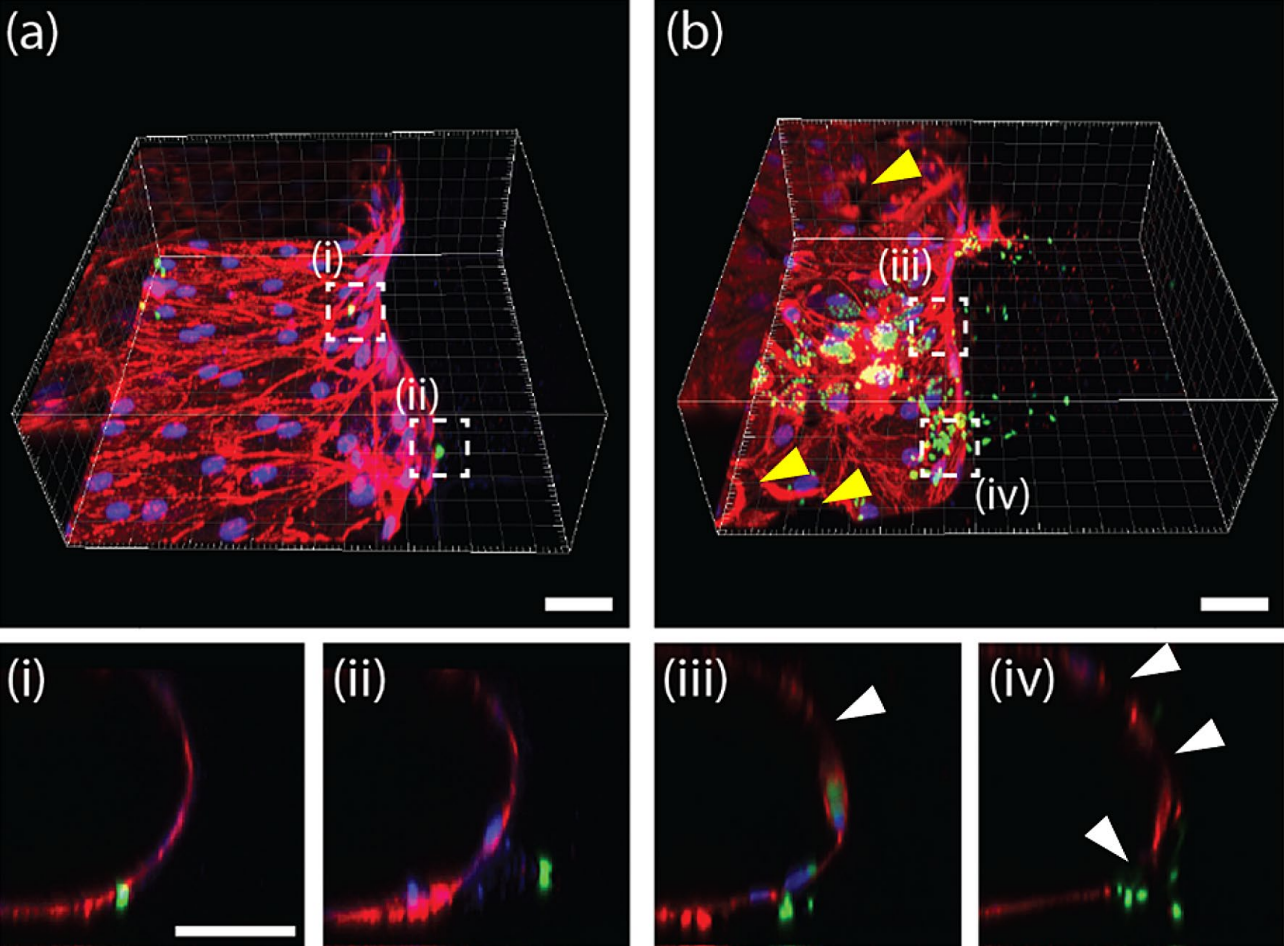
Tachyzoites (GFP (green)) transmigrating the EC monolayer (rhodamine-phalloidin (red) and DAPI (blue)) at (**a**) 12 h and (**b**) 60 h after infection. The image in (i–iv) at the bottom indicate a cross sectional view of the white box in (**a,b**). Tachyzoites were (i) either trapped in the EC monolayer or they (ii) migrated into the ECM after transmigration. Another image showing the coincident (iii) transmigration and proliferation (in an EC) of tachyzoites. (iv) Following egression, tachyzoites invade the ECM after disrupting the EC monolayer. Scale bar indicates 50 μm.

To reproduce the *T.gondii* infection to EC monolayer (Fig. 1a), we used a previously reported microfluidic model with 3D EC monolayer. As demonstrated previously, the reconstituted EC monolayer on the rBM above the type 1 collagen hydrogel was found to be 3D, confluent (Fig. 2a–c), and of low permeability^44^. Tachyzoites were seeded at a density of 2 × 10^6^ cells/ml on the EC monolayer and incubated at 37 °C for 4 h, to induce microneme protein^48^ mediated adhesion. The medium in the channels was refreshed every 12 h, during which time non-adherent tachyzoites were removed. The Lytic cycle of the tachyzoites in the EC monolayer, including infection, replication, proliferation, and egress was monitored (Fig. 2d–f). The tachyzoites proliferated in the infected ECs for 36 h before actively egressing from the host ECs and migrating through the ECM to invade the adjacent ECs. The normalized infected area increased rapidly after 36 h, indicating that the in vitro cycle (of infection to egress) was approximately 36 h, as previously reported^49^. Up to 60 h, the normalized increase rate of the infected area fraction was greater than 2.2 times per 12 h. However, the rate of area increase begins to slow at 72 h, peaks at 84 h, and then decreases at 96 h. Since the majority of the surrounding cells were damaged or dead, the egressed *T. gondii* was washed away by the flow when the culture medium was changed (Fig. 2e). The sequential steps of tachyzoites and ECs interaction were captured in a single image (Fig. 2f). In the case of uninfected or recently infected ECs, the junction remained intact and F-actins were evenly distributed around the nucleus (Fig. 2f(i)). After replication, the ECs became spherical and the intercellular spaces widened (Fig. 2f(ii)). During egression, the ECs disappeared (Fig. 2f(iii)). Some tachyzoites directly penetrate the EC monolayer during the early stages of infection (12 h after infection) (Fig. 3a(i–ii)). This penetration is clearly distinct from general mechanism, infecting ECs (Fig. 3b(iii)), migrating into the ECM after egress (Fig. 3b(iv)), and causing partial damage of ECs (white arrows) with a gap observed (yellow arrow) (Fig. 3b(iv)). This experiment cannot determine conclusively whether tachyzoite transmigration is paracellular or intracellular. Previous studies that cultured 2D EC monolayers in petri dishes or transwells^18,20^ could not make these observations.

### 3D migration of *T. gondii* from the EC monolayer co-cultured with neurons

**Figure 4:**
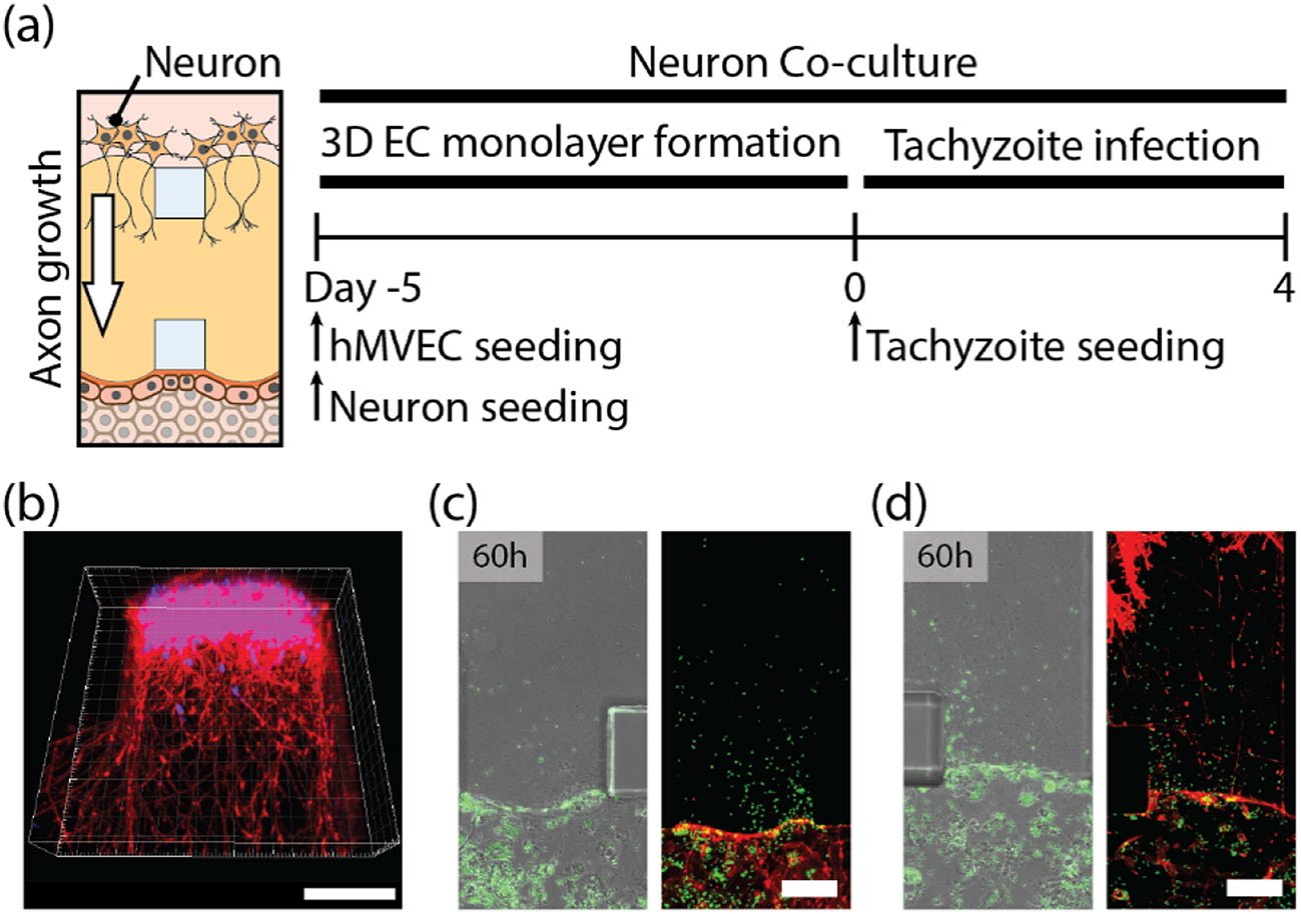
Tachyzoite infection of the EC monolayer co-cultured with neurons. (**a**) Time line for EC monolayer formation (with neurons) and tachyzoite infection. (**b**) Confocal image of neurons (rhodamine-phalloidin (red) and DAPI (blue)) on ECM shows the 3D migration of axons into ECM. Fluorescent images after 60 h of tachyzoite infection (**c**) without and (**d**) with neurons. Scale bars indicates 100 μm.

**Figure 5:**
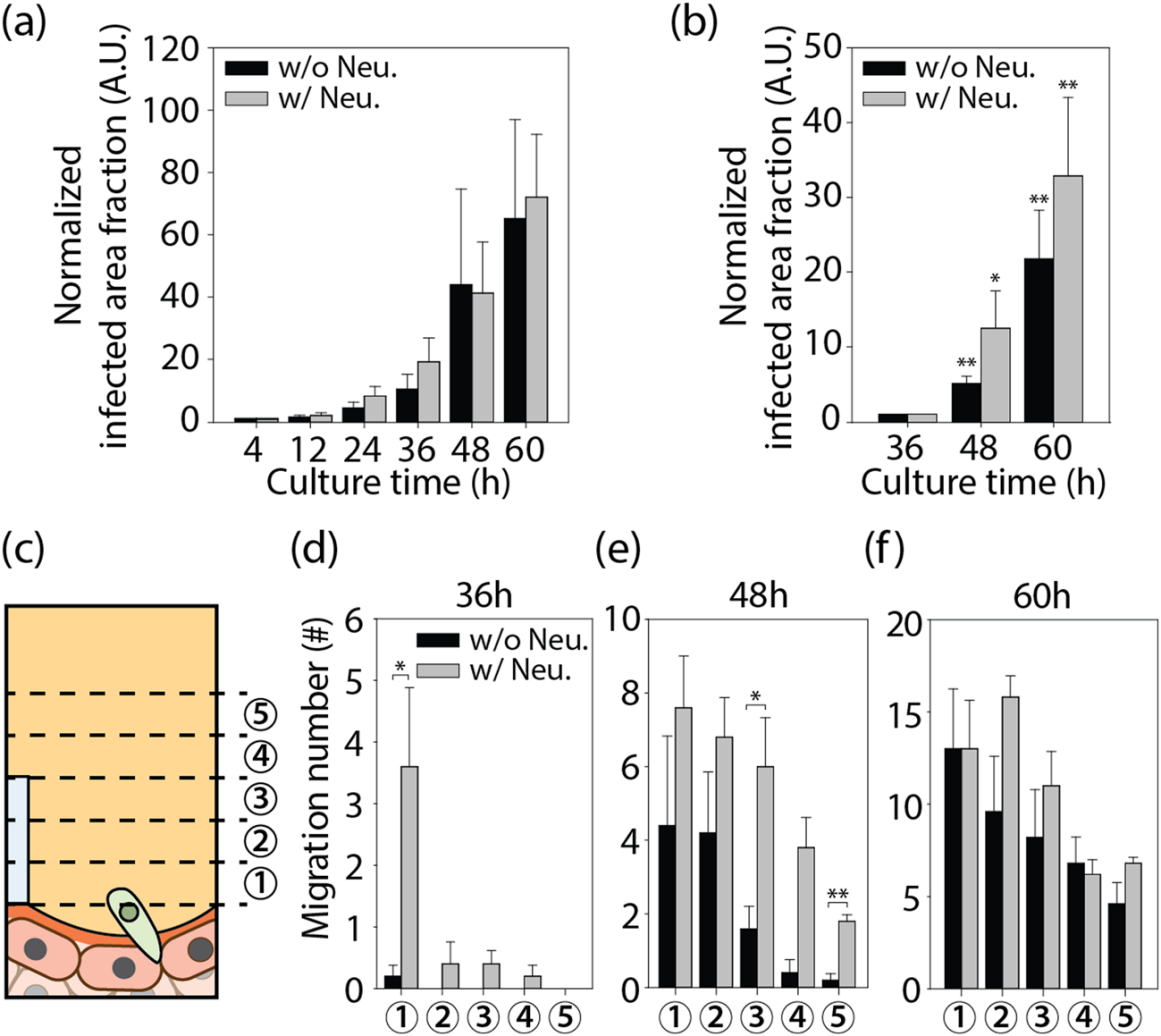
Tachyzoite area change (**a**) on EC monolayer and (**b**) into the ECM when cultured with or without neurons. (**c**) Quantification of the tachyzoite invasion of the ECM. (d-f) Number of individual tachyzoites in each region of interest at 50 μm intervals after 36, 48, and 60 h of tachyzoite infection (n = 5 for each group). Data are presented as averaged ± SD. Statistical significance was analyzed for panels (**b**) and (**d–f**) by one-way ANOVA with Holm-Sidak method and is indicated by asterisks as follows: *P < 0.05, **P < 0.01, ***P < 0.001.

A microfluidic protocol for the co-culture of an EC monolayer with adjacent neurons was developed to model the encysted parasites that are commonly found in the brain during chronic infection^50^. Neurons were seeded on one side of the ECM to facilitate the 3D growth of axons in the ECM. After rBM coating, ECs were seeded on the opposite side of the ECM, and after 5 days, a confluent monolayer formed (Fig. 4a). After 5 days, neurons extended axons into the ECM (Fig. 4b). After a 36-h lytic cycle, tachyzoites infected the confluent EC monolayer and moved directly into the ECM (Fig. 4c,d). Notably, co-culturing neurons did not increase the number of tachyzoites in the EC monolayer, but it did increase their migration into the ECM (Fig. 5a,b). Figure 5c,d depict the egress of tachyzoites into the ECM when neurons are cultured, as indicated by the increased number of tachyzoites near the EC monolayer at 36 h. At 60 h, tachyzoites cultured in the absence of neurons began to egress from the ECs and reach a similar population in the ECM (Fig. 5e,f). Co-culturing tachyzoites with neurons appeared to enhance their invasion into ECM, but to have no effect on the invasion-egress cycle or the number of tachyzoites on the EC monolayer. Tachyzoites preferred to migrate out of neuron co-cultured EC monolayer, to initiate cycles of duplication, proliferation, and egress within the ECs. After 48 h, the migration of tachyzoites towards the neurons slowed down. According to a previous study, tachyzoites are co-localized with neurons, transform into bradyzoites and form brain cysts^10,51^. Previous studies have reported the presence of in vivo cysts in the neuron soma of the brain^52,53^ and the possibility of encephalitis^54^. In our experiments, we were unable to detect bradyzoites, tachyzoites infected neurons, or the formation of cysts (Supplementary Fig. 1).

### Effect of chemicals on *T. gondii* infection, egress, and transendothelial migration

**Figure 6:**
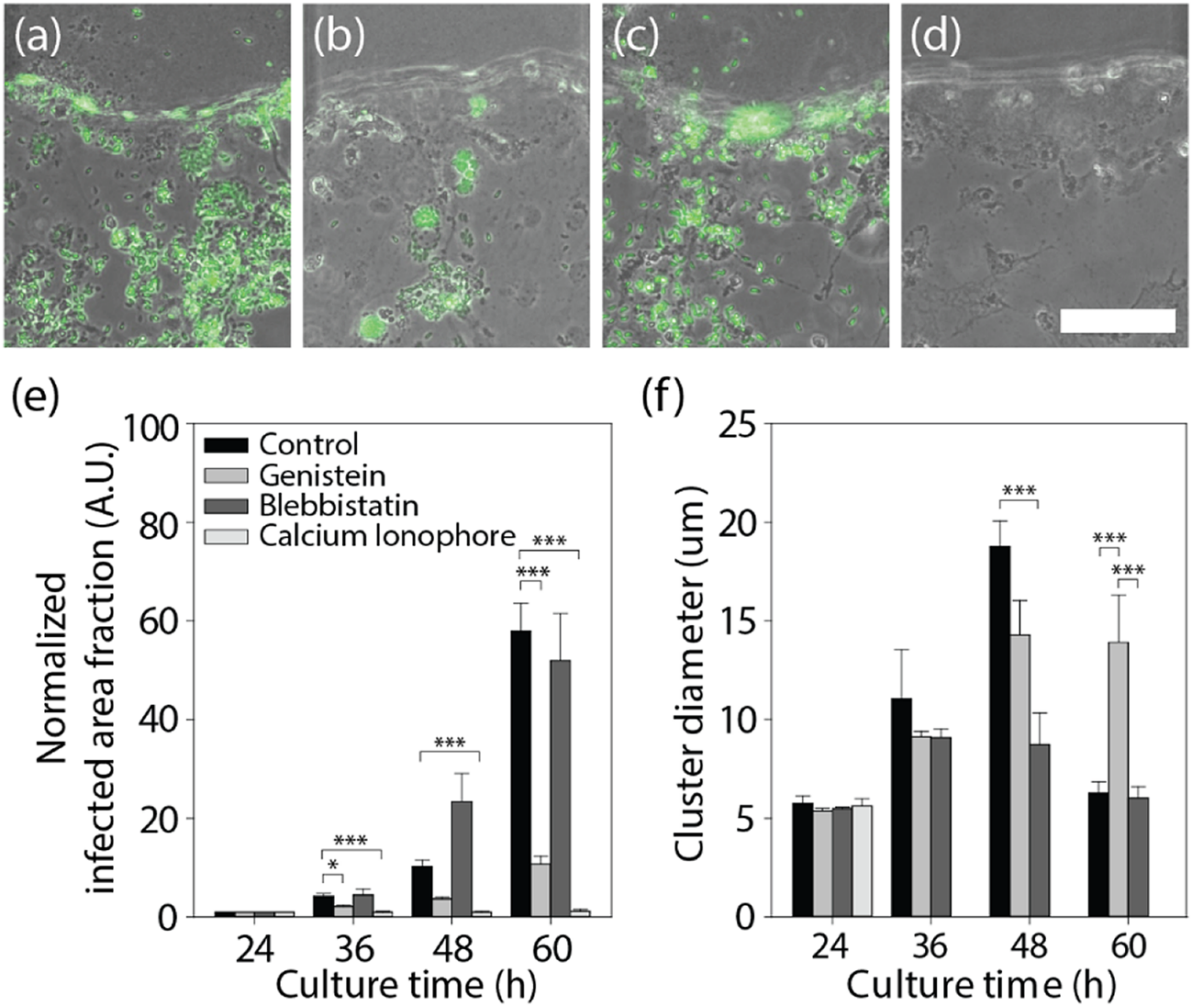
Chemical treatment of tachyzoites infecting the EC monolayer. (**a–d**) Images after 60 h of processing each chemical; (**a**) control, (**b**) Genistein, (**c**) Blebbistatin, and (**d**) calcium ionophore. (**e**) Proliferation of tachyzoites in the EC monolayer, and (**f**) their cluster size. Scale bar indicates 100 μm. Statistical significance was analyzed by one-way ANOVA with Holm-Sidak method and is indicated by asterisks as follows: *P < 0.05, **P < 0.01, ***P < 0.001.

In the microfluidic assay, three chemicals were evaluated: a tyrosine kinase inhibitor (genistein)^45^, a myosin II inhibitor (blebbistatin)^55^, and a calcium ionophore (A23817)^47^ (Fig. 6a–d). Each chemical was added to the medium and refreshed every 12 h until the end of experiment. The tachyzoite-infected area fraction and cluster size within infected cells were measured also every 12 h (Fig. 6e,f). It was discovered that both genistein and calcium ionophores significantly reduced the overall infection caused by tachyzoites. Only genistein, however, maintained tachyzoite proliferation within infected ECs. The ability of kinase inhibitors to block the effect of calcium ionophores on the egress of tachyzoites from host cells, such as kidney cells^55^ or neutrophils^56^, has been reported in prior researches. Using a microfluidic chip, we evaluated tyrosine kinase inhibitors to prevent the spread of *T. gondii* for the drug candidates of previous papers on *T. gondii*. Our experiments confirmed that microfluidic chips could be a promising platform for drug screening with parasitic infections. The tyrosine kinase inhibitor could be a promising target for the development of anti-*T. gondii* therapies. Calcium ionophore A23817 is known to inhibit host cell invasion and the intracellular replication of tachyzoites^57^, in addition to enhancing the egress of tachyzoites from host ECs prior to proliferation^47,58^. Experiments confirmed that the infected area fraction and cluster size of the tachyzoites decreased drastically as a result of the calcium ionophore's strong inhibitory effect on tachyzoite replication in host cells. However, A23817 demonstrated severe toxicity in ECs, which may restrict the drug's application. It is known that blebbistatin inhibits the actomyosin motor, myosin II, which forms bipolar filaments that constitute the contractile array by interlocking with actin filaments, thereby inhibiting ionophore-induced egress^46^. Nonetheless, blebbistatin was discovered to facilitate the egress of tachyzoites from their host ECs prior to complete proliferation. This finding supports the notion that myosin II also plays a role in tachyzoite egress from host cells, similar to its role in virus egress^46,59^.

## Discussions

We replicated 3D tachyzoite infection of EC monolayer adjacent to neurons in a microfluidic chip. Multiple days of *T. gondii* lytic cycle could be successfully monitored. *T.gondii* was able to easily infect EC monolayer, which contained their proliferation. *T.gondii* egressed from the infected EC monolayer and migrated through the ECM towards neurons. The inability to demonstrate neuronal infection of tachyzoites is one of the limitations of our research. Migration of tachyzoites was enhanced by the presence of neurons on the opposite side of the ECM, possibly due to neuron-secreted substances. Physical interaction between tachyzoites and axons could not account for the enhanced migration. Throughout their invasion, tachyzoites did not align with axons. Axons grew over only half of the ECM hydrogel, not reaching the EC monolayer; however there was a dramatic increase of tachyzoites around the EC monolayer 36 h after infection (Fig. 5d). The dramatic trans-endothelial migration may be attributable to the stiff gradient of enriched neuron-secreted substances over the EC monolayer. In our studies, tachyzoites did not infect neurons directly. For the reconstitution of advanced clue for the infection, i.e. cyst formation in brain, a complex cerebral microenvironment may be required, which could be realized with cerebral organoids in future research.

Through drug screening experiments (Fig. 6), the precise mechanism of anti- *T. gondii* drugs including A23187, Genistein and belbbistatin could be verified. A23187 was found very effective at eliminating *T. gondii*, but also caused previously unreported severe EC damage. The mechanism by which A23187 inhibits the egress of *T. gondii* appears to be completely distinct from that of genistein. The death of host cells can inhibit *T. gondii* proliferation, but also causes severe stromal damage. Additional in vitro experiments are required to determine the optimal concentration for pathogen suppression with minimal toxicity. The balance could be monitored using the developed model, which could be beneficial for the development of new anti-pathogenic drugs, considering effect on the microenvironmental factors. This in vitro study enables sequential real-time observations of the *T. gondii* lytic cycle, which was previously only confirmed in mouse model with expensive two- or multi-photon microscopy^60,61^. The developed method could be integrated with existing organoid technology as an advanced infection model with microenvironment^62^. Spatial segmentation of various microenvironmental components, including blood vessels, ECMs and neurons, was reconstituted, for the temporal application of a pathogen, *T.gondii* (Supplementary information).

## Supplementary Information

Supplementary Figure 1.

## Data Availability

The datasets used and/or analysed during the current study available from the corresponding author on reasonable request.
