# Supplementary material for: Microfluidic model for in vitro acute Toxoplasma gondii infection and transendothelial migration
Source: Sci Rep. 2022 Jul 6;12:11449. doi: 10.1038/s41598-022-15305-4 (PMC9259589; doi:10.1038/s41598-022-15305-4)
Supplement: Supplementary file 1 — Supplementary Figure 1. [file 41598_2022_15305_MOESM1_ESM.docx]

**Supplementary Information**

**Microfluidic model for *in vitro* acute *Toxoplasma gondii* infection and transendothelial migration**

*Hyunho Kim^1,2+^, Sung-Hee Hong^3+^, Hyo Eun Jeong^1^, Sewoon Han^4^, Jinchul Ahn^1^, Jin-A Kim^1^, Ji-Hun Yang^5^, Hyun Jeong Oh^1,^*********, Seok Chung^1,6,^*, Sang-Eun Lee^3,^**

^1^ School of Mechanical Engineering, Korea University, Seoul, Republic of Korea

^2^ Center for Systems Biology, Massachusetts General Hospital Research Institute, Boston, MA, USA

^3^ Division of Vectors and Parasitic Diseases, Korea Diseases Control and Prevention Agency, Cheongju, Republic of Korea

^4^ CellFE, Alameda, CA, USA

^5^ Next & Bio, Seoul, Republic of Korea

^6^ KU-KIST Graduate School of Converging Science and Technology, Korea University, Seoul, Republic of Korea


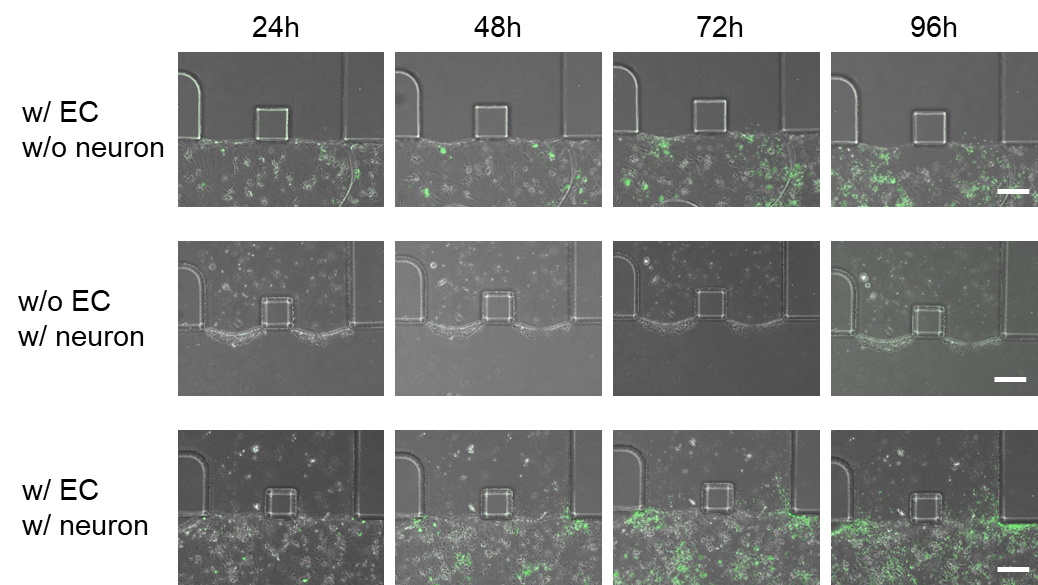


Supplementary figure 1. Infection with GFP-expressing toxoplasma in the presence or absence of neurons, as visualized by phase-contrast and green fluorescence images. Green: Toxoplasma, 150 μm Scale Bars.
